# Perspectives of future pharmacists on the potential for development and implementation of pharmacist prescribing in Qatar

**DOI:** 10.1007/s11096-019-00946-9

**Published:** 2020-01-02

**Authors:** Mohammad Issam Diab, Angham Ibrahim, Oraib Abdallah, Alla El-Awaisi, Monica Zolezzi, Rwedah Anwar Ageeb, Wishah Hamza Imam Elkhalifa, Ahmed Awaisu

**Affiliations:** 1 College of Pharmacy, QU Health, Qatar University, P.O. Box 2713, Doha, Qatar; 2 Mental Health Services, Hamad Medical Corporation, Doha, Qatar

**Keywords:** Mixed-methods, Pharmacist prescribing, Pharmacy graduates, Pharmacy students, Qatar

## Abstract

*Background* Pharmacists in many developed countries have been granted prescribing authorities under what is known as “non-medical prescribing” or “pharmacist prescribing”. However, such prescribing privileges are not available in many developing countries. *Objective* The objective of this study was to determine the perspectives of future pharmacists (recent pharmacy graduates and pharmacy students) on pharmacist prescribing and its potential implementation in Qatar. *Methods* A convergent parallel mixed-methods design was used: (1) a cross-sectional survey using a pre-tested questionnaire and; (2) focus group discussions to allow for an in-depth understanding of the issue, with a focus on pharmacists prescribing competencies as well as barriers for its implementation. *Main outcome measures* Future pharmacists’ perspectives and attitudes towards pharmacist prescribing in Qatar. *Results* The majority of the respondents (94.4%) indicated awareness of the prescribing competency related to selecting treatment options. Furthermore, the majority (92.4%) believed that pharmacists should undergo prescribing training and accreditation before been legally allowed to prescribe, a point that was reiterated in the focus group discussions. Participants generally expressed support for collaborative and supplementary prescribing models when developing prescribing frameworks for Qatar. Four categories emerged under the theme barriers to implementation of pharmacist prescribing: lack of prescribing competency, pharmacist mindset, lack of accessibility to patient records and counseling rooms, and diversity of education and training background. *Conclusion* The majority of recent pharmacy graduates and students were in favor of pharmacist prescribing been implemented in Qatar. However, a special training program was deemed necessary to qualify pharmacists to prescribe safely and effectively.

## Impact on practice

- Further changes to the pharmacy curriculum and pharmacist prescribing training program in Qatar are warranted in order to improve pharmacists’ competence in prescribing practice prior to its implementation.
- Prescribing models such as collaborative and supplementary prescribing are the most supported models and should be considered when developing prescribing frameworks for Qatar.
- Barriers to implementing pharmacist prescribing in Qatar must be addressed by various stakeholders to advance pharmacy practice and further optimize the utilization of pharmacy workforce.

## Introduction

In recent years, healthcare systems have been rapidly advancing with an increased focus on optimizing healthcare quality, ensuring safety, and strengthening the provision of patient-centered care [1, 2]. In parallel with the globally evolving healthcare needs, there has been an apparent expansion of pharmacy practice beyond the traditional technical and dispensing paradigm [1–4]. Owing to pharmacists’ role expansion and enhanced clinical training and professional expertise in evidence-based drug therapy management, they are considered as an integral part of healthcare teams [1, 2, 5, 6]. In addition to their involvement in medication therapy management, proactive patient counseling, and direct patient care, an evolving role for pharmacists is involvement in prescribing activities with varying degree of privileges [3–8].

Traditionally, the right to prescribe medicines was restricted to physicians and dentists only [8, 9]. However, over the past three decades, new policies and legislations have emerged to allow non-medical prescribing (NMP) [6, 10]. NMP is the legal right to prescribe medicines granted to healthcare professionals (HCPs) other than doctors and dentists, who have undergone a specified training and have attained appropriate competencies and qualifications to become prescribers [6, 8–11]. Pharmacists are among the range of other HCPs who have been given the right to prescribe in a number of developed countries, including the United Kingdom, the United States of America, Canada, Australia, and New Zealand [6, 7, 10, 12–14]. However, the level of authority to prescribe may vary in different areas, in that legal restrictions could apply on who can prescribe medicines, what they can prescribe, and whether they are allowed to do so independently [6–9, 15–18]. For example, in Canada, Alberta was the first province to authorize independent pharmacist prescribing for registered pharmacists in 2007 [7]. Most pharmacists in other Canadian provinces pursued prescribing activities under collaborative protocols before the legislation of such privileges across Canada in following years [7, 18–20]. Notably, the most common NMP models described in the literature include independent, dependent, and collaborative prescribing [6, 8, 10, 11, 17, 21–23]. The independent prescribing model grants a non-medical prescriber (NMPer) the sole responsibility of assessing, diagnosing, and making initial treatment decisions, while dependent NMPers are restricted in their activities by protocol/formulary [8, 11]. Meanwhile, collaborative prescribers are granted the legal authority to prescribe medications under a collaborative relationship towards achieving a set of agreed patients’ outcomes [6, 8, 11].

Essentially, the pharmacist prescribing initiative is aimed at improving the quality and continuity of patient care, improving medication management, improving patients’ access to medicines and healthcare, saving time for patients and medical practitioners, and improving the utilization of resources by making better use of professional skills and expertise of the pharmacist [4, 9, 10, 15]. A recent systematic review of 45 studies, demonstrated positive outcomes in the management of acute and chronic diseases in favor of pharmacists and nurses NMP against medical prescribers (MPers) [23]. Such outcomes included improvements in lipid profiles, glycated hemoglobin, and blood pressure levels. Additionally, the meta-analysis of 14 studies showed that 85% of patients were generally satisfied with pharmacist-interventions [23]. Likewise, much of the current Canadian literature pertaining to pharmacist prescribing activities has demonstrated significant evidence of improvements in similar clinical parameters [6, 24–29]. A number of stakeholders also reported that prescribing practices by pharmacists were considered safe and effective [6, 30]. Furthermore, physicians, patients, and NMPers have reported that NMP by nurses and pharmacists reduced the workload of MPers, improved the continuity of care, and increased job satisfaction and professional confidence [9, 10, 15, 30].

In addition, suboptimal medical prescribing has also been evident in the literature, suggesting the potential impact of NMP on reducing medication errors [31–36]. Studies assessing prescribing competencies of different healthcare students (e.g. medical, pharmacy, and nursing students) are scarce. One comparative study found that pharmacy students had better knowledge of basic pharmacology than medical students (77.0% vs. 68.2%; *P* < 0.001), yet both groups had similar knowledge of applied pharmacology (73.8% vs. 72.2%; *P* = 0.124) [35]. Another study also showed that pharmacy students had a significantly higher identification rate of prescription errors than that of medical students [36]. In addition, evidence shows that pharmacists’ earlier involvement in the prescribing process may help optimize the use of medicines [37, 38].

Currently, pharmacist prescribing is considered a novel area of practice in Qatar and the Middle East. Nonetheless, the need for expanding the scope of pharmacy practice/services, including prescribing, corresponds with the new launch of Qatar’s National Health Strategy 2018–2022 [39]. Accordingly, it is important to determine the views, perspectives, and experiences of all stakeholders—including pharmacy students—about prescribing competencies and the potential implementation of pharmacist prescribing in Qatar. Little is known about the perspectives and views of pharmacy students on prescribing competencies and implementing pharmacist prescribing. Only two studies were identified that investigate pharmacy students’ perspectives on implementing NMP and acquisition of prescriptive authority [17, 40]. Despite the diversity in students’ views and experiences, students in both studies expressed their preparedness to undertake prescribing role. However, differing views among future pharmacists may arise due to the variability of laws, policies and priorities in different regions [17]. To our knowledge, there were no previous studies conducted in this regard in Qatar or in the Middle East. This study is part of a larger project (Pharmacist Prescribing in Qatar Project) in which the investigators look at various stakeholders (including pharmacists, pharmacy educators, pharmacy students and recent graduates, academic leaders in health professional education, health policymakers etc.) who may have a potential role in the development and implementation of pharmacist prescribing in the State of Qatar.

## Aim of the study

The aim of this study was to determine, and explore in depth, the perspectives and attitudes of pharmacy students and recent pharmacy graduates towards pharmacist prescribing as an emerging role for pharmacists in Qatar. This study along with other studies from our larger project will provide foundational basis for implementing an evidence-based practice, which addresses national health priorities that are aimed at improving efficiency in the healthcare system and improving access to medicines.

## Ethics approval

The study received ethics approval from Qatar University (QU) Institutional Review Board (approval reference number: QU-IRB924-EA/18). The study did not involve any medical interventions or invasive procedures. Completing the web-based survey was an implied consent to participate in the cross-sectional survey. Informed consent forms were obtained from those who participated in focus group discussions.

## Methods

### Study design and setting

This study was conducted at the College of Pharmacy (CPH), QU. A convergent parallel mixed-methods design was used, where both quantitative and qualitative data were independently and concurrently collected to increase the validity and comprehensiveness of the data [41–43]. This would allow complementarity of both quantitative and qualitative methodologies and an in-depth understanding of the research question. Below is a detailed account of the two components.

### Quantitative component: cross-sectional survey

#### Study sample and sampling technique

All current pharmacy students in the CPH at QU (BSc, PharmD, and MSc in Pharmacy Practice) and recent pharmacy graduates (BSc graduates of the previous academic year who were not currently in the MSc or PharmD program at QU CPH) were invited to participate in the study (n = 146). All part-time PharmD and MSc students who did not receive their BSc at QU, in addition to recent graduates who were already employed, were excluded from the study. Universal sampling technique was followed in view of the low number of study population.

#### Survey instrument development

An anonymous, self-administered link using SurveyMonkey^®^ online software (SurveyMonkey^®^, San Mateo, CA, USA) was designed and then circulated to participants via emails. The questionnaire used was constructed based on the objectives of the study and through an extensive literature review. The three-main utilized key published literature related to prescribing were “Competencies required to prescribe medicines” by National Prescribing Service at Australia and the Prescribing Competency Framework by Royal Pharmaceutical Society, in addition to the “Guide To Good Prescribing: A Practical Manual” by World Health Organization (WHO) [44–46]. Other pertinent studies around similar objectives were also used for developing the questionnaire [47–54].

The questionnaire was divided into five sections: (1) Participants’ demographics; (2) Awareness regarding pharmacist prescribing and prescribing competencies; (3) Beliefs and views about prescriptive authority; (4) Perceptions regarding prescribing competencies covered in QU-CPH curriculum; (5) Barriers and facilitators to implementation of pharmacist prescribing in Qatar.

All items were developed to adapt the culture and setting. The type of questions varied according to the purpose. Multiple answer questions were used for demographics section, barriers and facilitators. Close-ended questions such as (Yes/No/Not sure) were used for awareness section and items anchored on a five-point Likert scale were used for beliefs and views about prescribing authority and for perceptions regarding prescribing competencies covered in curriculum. Open ended questions were used as required. The questionnaire underwent content validity by three pharmacy faculty members with expertise in pharmacy practice research and survey instrument development, specifically with experience in NMP research. Modifications were done to the draft of the questionnaire through an iterative process. Then, the questionnaire was piloted using a sample of five recent graduates, who were not eligible to participate in the study, to determine clarity, readability, and comprehensiveness of the developed items. Minor modifications were made before the final version of the questionnaire was made available.

### Qualitative component: focus groups discussions

#### Sampling and recruitment

Students and recent graduates who participated in the quantitative phase of the study were invited to take part in focus group discussions (FGDs) to allow for an in-depth understanding of the issue, with a focus on pharmacists prescribing competencies and inclusion of content in pharmacy curriculum or development of continuing professional development courses related to pharmacist prescribing. Each focus group session compromised 6–7 participants and lasted for about 60–90 min. The concept of theoretical saturation was followed. After four focus groups, no novel themes were emerged, and saturation was judged to be reached.

#### Data collection

A topic guide for the focus group discussions was developed based on the same literature review used in Phase 1. The guide was developed by two researchers and tested for validity by five other research members. Each focus group session was audiotaped using a digital audio recorder. Each session was transcribed verbatim with consideration to non-verbal expressions. A thematic approach of data analysis was used, and a framework was generated which allowed for structuring, labeling and defining data. Each phrase was coded with a code that reflects the meaning of the passage. Similar phrases, passages and ideas were sorted together under the same code and all these codes were used to generate themes.

#### Data analysis

For quantitative phase, statistical analyses were performed using Statistical Package for Social Sciences, version 24 (IBM SPSS^®^ Statistics for Windows; IBM Corp, Armonk, New York, USA). Frequencies and percentages were used to summarize the responses generated. For qualitative phase, thematic content analysis was used to structure the information derived from focus group discussions. Coding was done manually by three investigators with experience in qualitative research.

## Results

### Cross-sectional survey

#### Participants’ demographic characteristics

Of the 146 eligible students and recent graduates who were invited to participate in the study, 105 (72%) completed the online survey. About 28 (26.7%) were recent graduates, while the rest were students across all professional years. As shown in Table 1, around half of them (n = 54, 51.4%) were 21–23 years old. The majority of the participants (n = 55, 52.3%) were from Egypt and Sudan. About 38% of the participants have not had any experiential training (i.e. clinical rotations). Further details about the demographics of the participants are provided in Table 1.

**Table 1:** Future pharmacists’ demographic and academic characteristics (N = 105)

Parameters	n (%)
*Age*	
18–20 years	27 (25.7)
21–23 years	54 (51.4)
24–26 years	24 (22.9)
*Nationality*	
Qatari	10 (9.5)
Egyptian	39 (37.1)
Sudanese	16 (15.2)
Jordanian	10 (9.5)
Palestinian	5 (4.8)
Syrian	7 (6.7)
Others	18 (17.1)
*Current year in pharmacy college*	
First professional year (P1)	21 (20)
Second professional year (P2)	19 (18.1)
Third professional year (P3)	17 (16.2)
Fourth professional year (P4)	20 (19.0)
Recent BSc graduates of the previous year	28 (26.7)
*Number of clinical rotations completed so far during BSc and/or PharmD*	
0	40 (38.1)
1	20 (19.0)
2	1 (1.0)
3	1 (1.0)
4	1 (1.0)
5	0
6	29 (27.6)
More than 6	13 (12.4)

n, number of respondents

#### Awareness regarding pharmacist prescribing and prescribing competencies

**Fig. 1:**
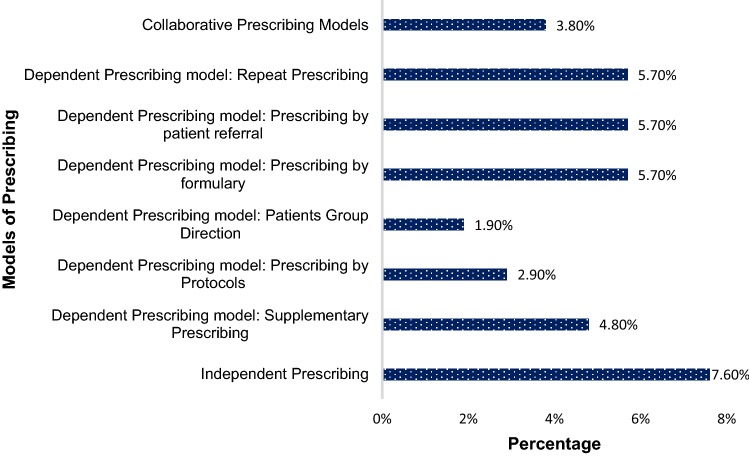
Future pharmacists’ awareness of international prescribing models

More than two-thirds of the participants (n = 76, 72.4%) had not observed the role of the pharmacist as a prescriber (either independently or through a protocol) in Qatar. Only 31 (29.5%) respondents expressed awareness (i.e. knowing and understanding) of at least one international prescribing competency framework. However, more than 66–95% of them expressed awareness of individual competency areas listed in the international framework as shown in Table 2. About 38% and 34% of the respondents were unsure or unaware of the prescribing competency related to prescribing professionally and improving prescribing practice, respectively. Only 8.6% of them were aware of the different models of NMP that are being implemented internationally. The most known prescribing model pointed by the participants was independent prescribing. Figure 1 illustrates the respondents’ awareness of international prescribing models.

**Table 2:** Students’ and recent graduates’ awareness of prescribing competencies (N = 105)

Statements	n (%)		
	Yes	No	Not sure
Assessing the patient (e.g. obtaining information to understand a person’s clinical needs, performing a comprehensive medicines assessment, generating and exploring possible diagnosis)	94 (89.5)	4 (3.8)	7 (6.7)
Considering treatment options (e.g. identifying and discussing appropriate, safe, effective, and evidence-based treatments for the patient)	100 (95.2)	4 (3.8)	1 (1.0)
Reaching a shared decision (e.g. negotiating therapeutic goals, reaching agreement about medicines to treat the person’s condition, and tailoring the treatment plan to meet the needs of the person)^a^	94 (90.4)	7 (6.7)	3 (2.9)
Prescribing medicine (e.g. prescribing with adequate, up-to-date awareness of medication actions, indications, dose, contraindications, interactions, cautions, and unwanted effects; prescribing within relevant frameworks for medicines use… etc.)	74 (70.5)	12 (11.4)	19 (18.1)
Providing information (e.g. providing information to other health professionals to ensure that the treatment plan is implemented safely and effectively)^a^	95 (91.3)	7 (6.7)	2 (1.9)
Monitoring and reviewing (e.g. obtaining and interpreting information to decide whether the therapeutic goals have been achieved whether to continue/stop treatment or refer the person to another health professional for further assessment)	94 (89.5)	4 (3.8)	7 (6.7)
Prescribing safely (e.g.: know and report prescribing errors, minimize risks, keep up-to-date with safety concerns)	93 (88.6)	6 (5.7)	6 (5.7)
Prescribing professionally (e.g. practicing in accordance with the relevant legislative, regulatory, professional, and organizational frameworks and applying quality use of medicines principles)	65 (65.7)	15 (14.3)	25 (23.8)
Improving prescribing practice (e.g. working to continually improve prescribing practice by reflecting and acting upon feedbacks, discussions, unsafe prescribing and understanding available tools to improve prescribing)	69 (65.7)	15 (14.3)	21 (20)
Prescribing as part of a team (e.g.: communicate and collaborate effectively with the person and other health professionals)^a^	81 (77.9)	10 (9.6)	13 (12.5)

^a^Missing data = 1

#### Beliefs and views of future pharmacists about prescriptive authority and implementations of pharmacist prescribing in Qatar

More than 90% of the participants believed that pharmacists can accurately adjust medication regimens to improve patient outcomes and that they should have an accredited prescribing training before being legally allowed to prescribe medicines. However, around one-fifth of the participants disagreed that pharmacist prescribing is likely to be accepted by patients. Overall, 57–97% of the participants agreed to all the statements in Table 3, except for one statement. There was less agreement to the statement “Physicians are likely to be in favor of prescribing by pharmacists”, where only 24.7% of the participants agreed to the statement.

**Table 3:** Beliefs and views of future pharmacists about prescriptive authority and implementation of pharmacist prescribing in Qatar (N = 105)

Statements	n (%)		
	Disagree	Neutral	Agree
1. I think pharmacist can accurately assess and diagnose patients within their competence^a^	11 (10.6)	26 (25)	67 (64.4)
2. Overall, I believe pharmacists can accurately choose and initiate the optimal drug therapy for a patient’s diagnosis	1 (1)	12 (11.4)	92 (87.6)
3. I believe pharmacists can accurately adjust a medication regimen to improve patient outcomes	0	5 (4.8)	100 (95.2)
4. I believe pharmacists should have prescribing training and accreditation or certification before being legally allowed to prescribe medicines within their competence	3 (2.9)	5 (4.8)	97 (92.4)
5. I believe all pharmacists should have prescriptive authority (i.e. legal authority to prescribe certain medicines)	22 (21)	17 (16.2)	66 (62.8)
6. Pharmacists are able to prescribe safely and effectively	2 (1.9)	11 (10.5)	92 (87.7)
7. Pharmacists are able to prescribe cost-effectively	6 (5.8)	10 (9.5)	89 (84.7)
8. Prescribing by pharmacists is likely to be acceptable to patients	20 (19.1)	25 (23.8)	60 (57.1)
9. Physicians are likely to be in favor of prescribing by pharmacists	57 (54.2)	22 (21)	26 (24.7)
10. Prescribing by pharmacists may potentially reduce medication errors in practice^a^	4 (3.8)	17 (16.3)	83 (79.8)
11. Pharmacist prescribing will increase better utilization of pharmacist’s skills and knowledge	3 (2.9)	5 (4.8)	97 (92.4)
12. Pharmacist prescribing will lead to improved patient access to medicines	3 (2.9)	14 (13.3)	88 (83.8)
13. Patients accessing prescribing by pharmacists will have better continuity of care	2 (2)	16 (15.2)	87 (82.9)
14. Prescribing authority for pharmacists will increase job satisfactions	3 (2.9)	8 (7.6)	94 (89.6)
15. Prescribing authority for pharmacists will increase self-confidence	1 (1)	5 (4.8)	99 (94.3)
16. Prescribing by pharmacists will reduce physician’s workload so they focus on more acute patients’ cases	5 (4.8)	16 (15.2)	84 (80)
17. Pharmacist prescribers must have access to the information in physician’s medical notes prior to prescribing	0	3 (2.9)	102 (97.1)
18. Pharmacist prescribers must be able to record prescribing actions in medical records^a^	0	4 (3.8)	100 (96.2)
19. I feel it is my professional duty and obligation to become a pharmacist prescriber upon graduation	8 (7.6)	14 (13.3)	83 (79.1)
20. Overall, I feel confident in my ability to become a pharmacist prescriber upon graduation	4 (3.9)	18 (17.1)	83 (79)

^a^N = 104

#### Future pharmacists’ perceptions regarding prescribing competencies covered in the current pharmacy curriculum with reference to WHO standards

With regards to the current curricula in the CPH, participants had differing perceptions regarding their preparedness with reference to WHO prescribing competency standards as shown in Table 4. Participants expressed preparedness to educate and provide instructions about the use of the prescribed medication (80.2%). About 82% of the participants agreed or strongly agreed to the statement: “Overall, I believe that I need special training and clinical experience on prescribing to be able to prescribe medicines”.

**Table 4:** Future pharmacists’ perceptions of prescribing competencies covered in the current pharmacy curriculum with reference to WHO prescribing competency standards (N = 101)

Competency standard	n (%)				
	Unprepared	Somewhat unprepared	Neutral	Somewhat prepared	Prepared
Assessing and defining patient’s problems (e.g. disease or disorder, sign of underlying disease, side effect of drugs, non-adherence to treatment)	4 (4)	5 (5)	10 (9.9)	35 (34.7)	47 (46.5)
Specifying therapeutic goals	5 (5)	1 (1)	4 (4)	24 (23.8)	67 (66.3)
Specifying alternative treatment (pharmacologic and non-pharmacologic)	4 (4)	1 (1)	6 (5.9)	16 (15.8)	74 (73.3)
Choosing a drug that is effective (by checking indication and convenience), safe (by checking contraindications, interactions and high-risk group) with low cost	2 (2)	3 (3)	8 (7.9)	18 (17.8)	70 (69.3)
Verifying suitable dose, route, dosage form, frequency and duration of drug for the patient	2 (2)	4 (4)	6 (5.9)	22 (21.8)	67 (66.3)
Writing a drug prescription independently	7 (6.9)	12 (11.9)	18 (17.8)	29 (28.7)	35 (34.7)
Educating and providing instructions about the use of the prescribed medication	2 (2)	2 (2)	6 (5.9)	10 (9.9)	81 (80.2)
Monitoring the outcome of drug therapy	3 (3)	4 (4)	8 (7.9)	26 (25.7)	60 (59.4)
Reviewing/altering prescription in the light of further investigations	3 (3)	6 (5.9)	13 (12)	20 (19.8)	59 (58.4)

N = 101 (missing four responses in this section)

#### Barriers and facilitators to implementation of pharmacist prescribing in Qatar

**Fig. 2:**
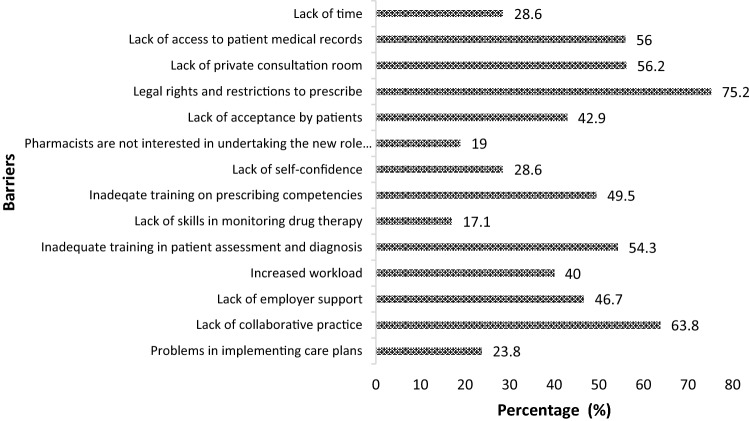
Future pharmacists’ perceived barriers to the implementation of pharmacist prescribing in Qatar

**Fig. 3:**
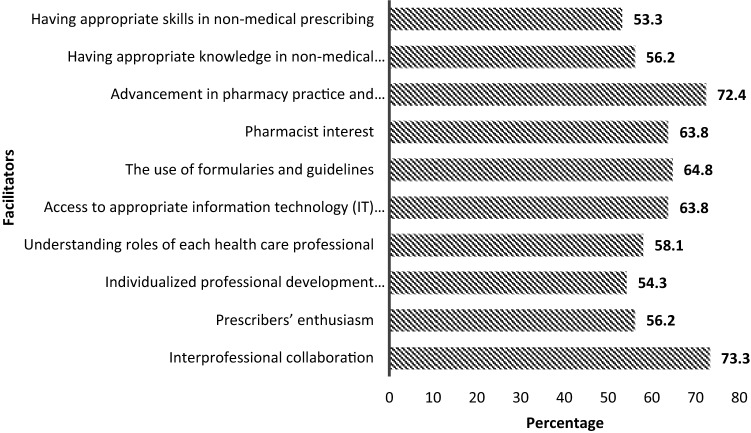
Future pharmacists’ perceived facilitators to the implementation of pharmacist prescribing in Qatar

Figure 2 illustrates the perceived barriers to the implementation of pharmacist prescribing in Qatar. Legal rights and restrictions to prescribe was the most commonly reported barrier (75.2%). This was followed by lack of collaborative practice (63.8%), lack of private consultation rooms (56.2%) and lack of access to medical records (56%). Lack of skills in monitoring drug therapy (17.1%) and pharmacist not interested in undertaking the new prescribing role were the least perceived barriers (19%). With regards to the facilitators, interprofessional collaboration was the most commonly reported facilitator (73.3%), followed by advancements in pharmacy practice and pharmacist’s role (72.4%) as shown in Fig. 3.

### Focus group discussions

#### Participants’ demographics and themes

Twenty-four future pharmacists consented to participate in the focus group discussions (FGDs). There were 4, 8, 3, 2 and 7 participants from P1, P2, P3, P4 and recent graduates, respectively. The mean (SD) duration for FGDs was 73 ± 17.3 min. Six main themes emerged from the FGDs. Table 5 represents a summary of the identified themes and their categories.

**Table 5:** Identified themes and subthemes/categories that emerged during focus group discussions

Themes	Categories
Benefits of pharmacist prescribing	Benefits to healthcare providers and system Benefits to pharmacy profession Benefits to patients
Barriers to implementation of pharmacist prescribing	Pharmacist incompetency Pharmacist mindset Lack of accessibility and support Pharmacist burden Diversity of education and training background
Level and model of prescribing	Knowledge of prescribing models Prescribing setting and restrictions Preferred models of prescribing
Facilitators of pharmacist prescribing in Qatar	Healthcare provider-related factors Pharmacist and pharmacy profession related-factors Patient-related factors Others: including governmental factors
Pharmacy curriculum	Participant’s awareness of prescribing guidelines Characteristics of current pharmacy program
	Gaps in the current pharmacy program
Implementation process	Characteristics of a training program
	Ways of implementation

#### Theme 1: benefits of pharmacist prescribing

Three categories emerged under the “benefits of pharmacist prescribing” theme. These are: benefits to HCPs and health care system (HCS), benefits to pharmacy profession and pharmacists, and benefits to patients as explained below.

Reducing burden, balancing tasks and saving efforts, reducing errors, prioritization of cases (minor vs. complicated) and better safe prescribing were all recognized as advantages for HCPs and HCS.

Many pharmacists have the ability to do minor diagnosis for some minor illnesses… So this will decrease the workload on the physicians (FGD2, X4)

… If pharmacists had more control over the prescribing process, these errors would not happen … So that would really keep the health centers to focus on patients who really need physicians (FGD 1, X3)

The participants have expressed that prescribing by pharmacist is an advantage for the profession and pharmacists themselves. Pharmacists will have better job satisfaction, enhanced confidence, better public image and better professional development. In addition, it will be an opportunity for pharmacists to apply their knowledge.

I think the huge advantage is that the public would be more aware of our role. If you are proving this opportunity, the public will view pharmacy like not a dispensing machine … the expectations will be much higher (FGD 4, X3)

Regarding benefits to patients, participants argued that pharmacist prescribing will save time for patients, offer them convenient and better care, and offer easy accessibility to HCS,

Better care provided for patients and again you know community pharmacies are really spread and they can find near to them so they can easily reach (FGD1, X1)

#### Theme 2: barriers to implementation of pharmacist prescribing

Five categories emerged under the theme “barriers to implementation of pharmacist prescribing”, including: lack of pharmacist competency in prescribing, pharmacist mindset, lack of accessibility and support, pharmacist burden and diversity of education and training background.

Lack of information and skills on diagnosis, lack of proper training and negligence of responsibilities are all incompetency of pharmacists highlighted by the participants. In addition, some participants stated that some pharmacists are incompetent when providing counseling to patients.

The pharmacists never looked at me and asked … he then gave me the medications. So, I mean this can be a disaster if we give such person the authority to diagnose and prescribe. (FGD1, X3)

For example, people from our college you see them they want to counsel and educate patients. However, you see people from outside who don’t have that? (FGD2, X4)

Additionally, pharmacist mindset has been perceived as a barrier to implementation of pharmacist prescribing. Disinclination, fear of additional load, inflexible habit of mind by pharmacists, and having a traditional social perception on the pharmacy profession were recognized as barriers that may hinder pharmacist prescribing.

Because of the training … physicians are taking the whole load … But I don’t think pharmacists they have this mentality that can go actually diagnose and prescribe … (FGD1, X2)

Lack of accessibility to patient records and counseling rooms, inability to order laboratory investigations, lack of support from physicians, public, and patients were also perceived barriers related to accessibility that support this subtheme.

…Lack of facilities also, such as physical structure of some settings. Counseling rooms are not available (FGD2, X3)

The patients and the physicians they will be resistant to the expansion of the role of pharmacists because they may feel threatened. (FGD4, X4)

Diversity of education and training background was an important subtheme under the barriers. Practice inconsistency, having pharmacist from different educational background, and variation in the role of pharmacists in community versus hospital setting were recognized.

I think that here in Qatar we have different pharmacy practitioners from different universities, not all of them have gone through the same thing (FGD 2, X5)

#### Theme 3: level and model of prescribing

Knowledge of prescribing models, prescribing setting and restrictions, and preferred models of prescribing were the categories that emerged under this theme.

Few participants were aware of the models implemented internationally. In Qatar, they have pointed that one known model is the collaborative agreement protocol that is being applied in the Heart Hospital, one of the Hamad Medical Corporation hospitals in Qatar.

[Pharmacist] can only change warfarin dose to increase or decrease… The physician will diagnose and tell that this patient is having heart failure…,and then the pharmacist will suggest the dose of warfarin … and adjust according to INR (FGD4, X5)

In relation to which model of prescribing suits the Qatar context, the majority agreed that the supplementary prescribing model would be the most suitable. Few interviewees suggested gradual implementation of pharmacist prescribing in Qatar.

I think supplementary [prescribing] would work better. I think the idea of having a shared care plan is a nice way to ease the pharmacists’ role in prescribing. Because if we just suddenly start with independent, it is going to make the stigma more and relationship will be more complicated between health care providers and pharmacists (FGD3, X4)

I think we can go with supplementary, make people who are independent prescribers be more adapt to the idea that is not all on you, we can share the responsibility and if they get to the idea or the rationale… If that could be installed on their heads, we can go into more collaborative practices (FGD4, X3)

#### Theme 4: facilitators of pharmacist prescribing

Four subthemes emerged as facilitators of pharmacist prescribing. These are factors related to: health care providers, pharmacist and pharmacy profession, patients, and others including governmental factors.

Participants have indicated that support should come by implementing regulations and policies and having a formal professional body (association) that speaks up for pharmacists.

I think we should have a pharmacy association or something or someone who can overlook the pharmacists and show that everything [including prescribing implementation] is going smoothly…. (FGD2, X8)

I think stakeholders and the governments, since they put rules and regulations, they should be the main support for the implementation of prescribing (FGD2, X6)

Additionally, facilitators of prescribing by pharmacists included pharmacy profession and pharmacists related factors such as capacity building and manpower development, providing training programs, authorizations to access patient information, and availability of competent pharmacists.

They [Pharmacists] are available so why not to use the manpower that you have (FGD1, X3)

With the college focusing on prescribing, the pharmacists will not only have the knowledge for prescribing but also will grow accustomed to this mentality (FGD1, X3)

#### Theme 5: pharmacy curriculum

Regarding participant’s awareness of prescribing guidelines, most of the participants were not aware. However, some of them believed that they heard about them, but never went through them in details. In addition, they have highlighted the characteristics of the pharmacy program at QU and stated that it is comprehensive, provide them with crucial knowledge and skills in a stepwise approach. Also, the program focuses on evidence-based practice which helps them in future as prescribers.

I believe one of the important skills that we learn in the undergraduate program is that we learn how to search and how to use guidelines and how to follow evidence-based practice. So that’s why maybe it is easier for us to know how to prescribe (FGD2, X3)

Furthermore, participants have highlighted that graduates from the PharmD program at QU are competent to prescribe.

I found them [QU PharmD graduates] very competent to prescribe and even they suggest the chemotherapy protocols (FGD2, X5)

However, some gaps in the pharmacy curriculum were reported. These included having some courses that are theoretical more than practical, insufficient courses related to patient assessment and diagnosis, and the need for additional preparatory courses that focus on prescribing.

I think we have gaps in terms of the materials related to diagnosing. We don’t take enough courses related to diagnosis of different diseases. We have only patient assessment. We take two courses and they are not enough. They are focused to certain things, but not to all related to human beings and also for the prescription, we didn’t take what a legal prescription should look like. (FGD2, X4)

#### Theme 6: implementation process

Two categories have emerged during the discussions related to the implementation of pharmacist prescribing in Qatar: (1) characteristics of training programs and; (2) the implementation approaches. A group of participants suggested that integrating prescribing in their undergraduate courses would help in implementing pharmacist prescribing in Qatar. Others agreed that it has to be a standalone program that is certified and accredited to allow graduates to become prescribers.

For QU students, definitely integration, because I think we cover a lot of aspects of prescribing in other courses. If we integrated into one of the courses, it will be less load to the student but for outside of QU [graduates] definitely should be a course or training (FGD4, X3)

For me, I don’t think the pharmacists really need a course, prescribing is just knowing the knowledge and applying it for choosing the drug. I don’t think it needs a whole course (FGD1, x2)

I think it is being done already in other countries like diploma so I believe we need to have the same thing here for six months for examples. (FGD2, X3)

## Discussion

The current study aimed to investigate the awareness, views, and attitudes of future pharmacists (pharmacy students and recent graduates) regarding the potential implementation of pharmacist prescribing in Qatar. Generally, the study participants had a poor awareness of internationally-recognized prescribing models. Nevertheless, the participants indicated that the pharmacy curriculum at QU-CPH has provided them with sufficient set of skills and knowledge pertaining to prescribing competencies. Yet, some participants highlighted the shortfall of the curriculum in detailing significant competency areas necessary to assume future prescribing roles. Such domains include diagnosing disease conditions and writing a prescription independently. Meanwhile, most study participants felt prepared to undertake roles in accordance with the prescribing competencies covered in the current pharmacy curriculum of the CPH at QU with reference to the WHO prescribing competency standards. Consistent with these findings, a study conducted in the UK demonstrated significant improvements in prescribing assessment scores (*P* = 0.007) and prescribing confidence (*P* = 0.0002) among final-year medical students following a doctor- and pharmacist-led prescribing teaching program, which was aimed at improving practical prescribing skills [55]. Previous studies among pharmacy students from Canada and Australia have also reported positive attitudes and confidence in undertaking pharmacist prescribing role [17].

Overall, most of the current study participants have highlighted the necessity of establishing special prescribing training programs to facilitate the acquisition of pharmacist prescriptive authority. Corresponding remarks pertaining to NMP implementation were reported in other countries by practicing pharmacists and other HCPs [12, 16, 30, 56–63]. Significant improvements in pharmacists’ knowledge were demonstrated through a competency assessment administered by the Saskatchewan government in Canada for pharmacists who intend to undertake independent prescribing roles for emergency contraption (ECP) services upon the completion of a specific training program for ECP prescribing [64]. It has also been reported that improvements in the educational structure could guarantee the generation of confident and skillful professionals instead of fearful and risk averting ones [12, 17, 56, 57]. Professional confidence could also be essential for proper practice implementation [65–67]. For instance, given the fact that many qualified NMPers do not carry out prescribing activities, the outcomes of implementing NMP are not yet clear. Thus, strategies of implementing NMP at different settings may neither be well evaluated nor a reliable reflection of real-world practice [11, 16, 17, 60–63, 65–67].

Differing views have emerged in the current study about applying training or education related to prescribing qualifications. While some participants were in favor of incorporating practical prescribing courses within the undergraduate degree curriculum, particularly those aimed at improving assessment and diagnosis skills, other participants were more inclined to establishing a standalone certified postgraduate program focused on NMP. Consistent with this, varying perspectives were reported in the literature regarding the inauguration of educational structures for the purpose of obtaining a NMP qualification [17, 57]. According to a 2013 study [17], students from an Australian university believed that a NMP qualification must be awarded through a post-graduate certification process. Conversely, their counterparts from a Canadian university suggested the inclusion of a set of specific training courses like a laboratory data analysis course aimed at achieving a successful prescribing culture in the undergraduate curriculum [17]. Interestingly, the undergraduate pharmacy curriculum at the CPH in QU covers such specific courses as ‘Patient Assessment Laboratory’ and ‘Interpretation of Clinical Laboratory Data’.

Further, the study participants disagreed that physicians will be in favor of pharmacist prescribing. One of the most common barriers highlighted by our focus group participants was the potential lack of support from other stakeholders such as physicians. According to the literature, the main barrier to implementing NMP is perceived to be the fear of physicians’ resistance due to impression of overlapping duties [56, 68–72]. Pharmacy students from other studies have also indicated that physicians’ backlash and blurring of the profession may influence the willingness of pharmacists to undertake prescribing roles [17, 40]. Further, it has been suggested that adopting a clearly defined scope of practice and recognizing the boundaries and limitations of the pharmacy profession could avert the issue of overlapping duties with physicians and other HCPs [17]. Another study that explored the perspectives of pharmacy students in Qatar on interprofessional education and collaborative practice, reported that pharmacy students were least confident about their professional identity [73]. This was attributed to lack of role models, existence of power and hierarchy between healthcare team members, and resistance to the evolving role of the pharmacists, especially by physicians [73].

The current study participants indicated the significance of receiving support through the amendment of laws and policies in favor of NMP. They have also identified interprofessional collaboration as a facilitator for implementing pharmacist prescribing. Similar perspectives and views were reported in the literature, whereby pharmacy students indicated the need for a well-established interprofessional experience aimed to increase the acceptance of pharmacists’ activities by other HCPs [17, 40]. Consistent with other research studies, most of the participants in this study considered supplementary and collaborative prescribing as the most suitable models to be implemented in Qatar [10, 17, 23, 40]. Particularly, future pharmacists believed that a gradual acquisition of prescriptive authority by pharmacists could help establish a trust culture between pharmacists and other HCPs and evaluate possible areas of improvements in pharmacists’ prescribing competencies.

This study was limited by a small sample size, which could affect the generalizability of the findings. However, given that there is only one school of pharmacy in the state of Qatar with an average of 25 students per professional year, the study has reported a high response rate. Another limitation of this study was the participants’ susceptibility to social desirability bias since the principal investigators were their professors. The pharmacy students and graduates maybe prone to cognitive bias or Dunning–Kruger effect by over polishing the image of their college and its curriculum. As a result, we have conducted a thorough curriculum mapping to better evaluate the quality and comprehensiveness of the curriculum of the CPH at QU with respect to prescribing-related content.

## Conclusion

The majority future pharmacy practitioners in Qatar were in support of implementing pharmacist prescribing. In addition, they have demonstrated their willingness to undertake prescribing roles in their future practices, particularly under a collaborative practice agreement. However, most participants recognized the need to establish focused pharmacist-prescribing training programs and to include relevant content in pharmacy curricula to further improve pharmacists’ practical prescribing skills prior to implementation. To further broaden the findings of the current study, future studies should investigate the perspectives of other healthcare students (e.g. medical, nursing, and dental students) on pharmacist prescribing and NMP in Qatar.
